# Efficacy of copper oxide nanoparticles using *Piper longum* and *Piper betle*

**DOI:** 10.6026/97320630019964

**Published:** 2023-09-30

**Authors:** Tejitha Ambati, Vamshi Nizampuram, Sahana Selvaganesh, Rajeshkumar S, Thiyaneswaran Nesappan

**Affiliations:** 1 Saveetha Dental College and Hospitals, Saveetha Institute of Medical and Technical Science, Chennai, Tamilnadu, India

**Keywords:** Efficacy, copper oxide, nanoparticles, *Piper longum* and *Piper betle*

## Abstract

It is of interest to evaluate the antibacterial, anti-inflammatory, and antioxidant effects of copper nanoparticles synthesized using *Piper longum* and *Piper betle*. The copper nanoparticles were characterized using various techniques and found to have a diameter between 30 and 90 nm. The nanoparticles exhibited significant antibacterial activity against *E. faecalis*, *S. aureus*, *C. albicans*, and *S. Mutans*, comparable to gold standards. They also demonstrated anti-inflammatory effects similar to the gold standard values. Furthermore, the copper nanoparticles displayed antioxidant capabilities, with maximum inhibition of 85.16% at 50 g/ml and a minimum inhibition of 50.62% at 10 g/ml. Overall, the study suggests that *Piper longum* and *Piper betle* mediated copper nanoparticles possess promising antibacterial, anti-inflammatory, and antioxidant properties, indicating their potential use in various applications.

## Background:

Nanotechnology is a novel and growing technology with several innovations in various fields. Nanotechnology is the art and science of developing nanoparticles, or materials with a size smaller than 100 nanometers [1]. Nanoparticles (NPs) serve as a link between bulk materials and atomic or molecular structures [2]. It revolutionized the fields of medicine and dentistry by improving the mechanical and physical properties of materials and aiding in the creation of innovative diagnostic procedures and nano-delivery systems. Nanoparticles are used in a variety of biological, diagnostic, and pharmacological applications, as well as in the administration of medications for the treatment of cancer and other infectious diseases [3,4].. The advancement of green synthesis using natural reducing, capping, and stabilizing agents without the need for hazardous, expensive chemicals and high energy consumption has drawn researchers to biological techniques [5,6,7]. Metal nanoparticles are becoming common because of their physical and chemical characteristics, large surface area for interaction, and wide range of applications [8]. Green synthesis of CuONPs has recently gained popularity due to the several benefits it provides over traditional physical and chemical approaches. The green chemistry method emphasizes the utilization of biological components for the production of CuONPs as a dependable, straightforward, and non-toxic method [9,10]. As a result, much emphasis is being placed on exploiting the production of CuONPs utilizing biological resources. Although there have been a few papers on the use of microbes and plant extracts for the synthesis of CuONPs [11,12,13]. Piper beetle and Piper Longum combined use have not been reported prior, and a review of the current literature revealed that this approach of CuONP synthesis has been less researched.

*P. Betle* and *P. Longum* Longum (Long Pepper) are small perennial creepers, belonging to the family Piperaceae. Piperaceae crops are widely farmed in India, Sri Lanka, Malaysia, Thailand, Taiwan, and other Southeast Asian nations [14, 15]. The primary components of these shrubs include volatile oil, carbohydrates, proteins, alkaloids, saponins, tannins, and mineral minerals (K, Mg, Na, Zn, Mn, and Co) necessary for human health, metabolic processes, healthy development, and lipid metabolism [16]. It is used to treat a variety of conditions including gonorrhea, cholera, chronic malaria, diarrhea, viral hepatitis, respiratory infections, stomachaches, coughs, bronchitis, tumors, and illnesses of the spleen [16]. It has anticancer, hepato-protective, antioxidant, antibacterial, anti-inflammatory, anti-dysenteric, anti-obesity, and anti-platelet effects [17, 19].

In dentistry, especially dental implantology, there is ongoing research on the various methods and feasibility of improving the closure of the flaps and tissues and ultimately maintaining the soft tissue health around the dental implants especially during the healing period. Even Though traditionally sutures have been in use for a prolonged period of time, sutures can be a source of accumulation of plaque and bacteria. Thus an innovative material that can be an effective tissue adhesive considering the oral environment and the saliva is a dire need. Cyanoacrylate gel is gaining popularity in this aspect, it lacks on the grounds of providing anti-bacterial and anti-inflammatory efficacy. Therefore, it is of interest to assess the *P. longum* and *P. betle* mediated CuO-NPs in terms of their structural, optical, vibrational, compositional, morphological, photocatalytic, and antibacterial capabilities for their incorporation as a neo sealing gel.

##  Material and Methods:

This research was carried out at the Gold Lab of Saveetha Dental College (SDC). This study was approved by the ethical committee of SDC. The analytical quality chemical reagents that were used in this experiment were all procured from SRL Chemicals.

## Green material synthesis:

**Figure 1:**
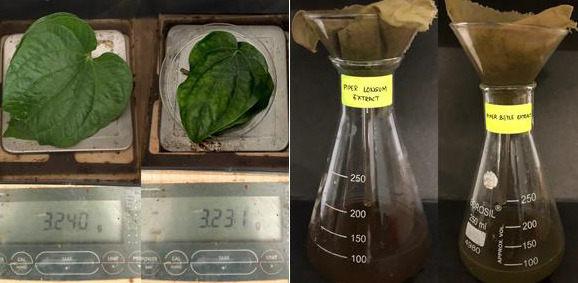
Showing the weight of *Piper longum* and *Piper betle* leaves taken and ground into fine paste and diluted and filtered.

Two medium-sized leaves of *Piper longum* and *Piper betle* weighing about 3.24 grams and 3.23 grams (Figure 1), respectively, were procured from the Herbal Garden of Saveetha Dental College, where they were produced under regulated circumstances and environments. After collecting the leaves, they were crushed into a fine mixture with a Mortar and Pestle. The obtained mixture was then diluted to 100ml by adding distilled water, and the plant extract solution was left to boil for 15-20 minutes to extract the phytochemicals, and then the concoction was filtered using a Wattman No. 7 Filter Paper.

## Synthesis of CuO nano-particles:

**Figure 2:**
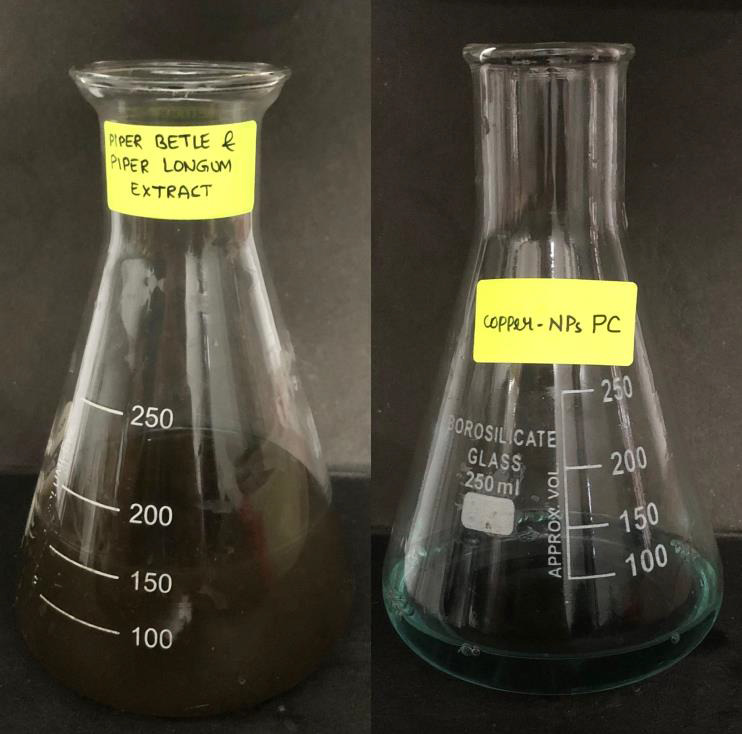
Showing the plant extract mixture, and copper nanoparticles precursor

After filtration, both filtrates were mixed together in a beaker to form plant formulation. Precursor solution was prepared by mixing 0.481g of CuSO4 with 50 ml of distilled water. Following that, 50 ml of precursor solution was mixed with 50 ml of the plant formulation and kept on a magnetic stirrer for 72 hours, at 600-700 rpm, Gradual color change was observed and the UV-Vis spectroscopy readings were taken, once every 24 hours to confirm the synthesis of Nanoparticles. A color shift was observed from dark green to black, and the agitation was maintained for 72 hours, (Figure 2) and then the sample was centrifuged at 8000 rpm for 10 mins. The pellet was collected and the supernatant was discarded.

##  Characterization of CuO nanoparticles

To characterize the produced CuNPs, the following techniques were used: UV-Vis spectrophotometer, FT-IR, SEM, and XRD.

## UV-Vis spectrophotometer:

The UV-Vis spectra of synthesized CuNPs were measured in the 250-650 nm region using a UV-Vis spectrophotometer (ELICO SL 210 UVVis spectrophotometer).

## FT-IR analysis:

Using an FT-IR spectrometer, the chemical binding of produced nanoparticles was investigated. The sample mixture was powdered and stored in a sterile Eppendorf tube using a heat treatment procedure. The powder was then utilized for FT-IR analysis by Attenuated Total Reflectance-Fourier Transform infrared spectroscopy (Bruker Alpha II FTIR) in the spectral region of 4000-550 cm^-1^ wavelength with 4 cm^-1^ resolutions at total scan of 64 scans per sample. Once the sample was exposed to radiation, a certain wavelength was absorbed by a particular chemical inside the sample, and a graph was created in accordance with this finding. Peak values were obtained that match the specific functional group.

## Scanning electron microscopy:

Carbon tape was used to mount the sample to the brass stubs. The sample was then placed in a vacuum chamber for Sputtering and then it was platinum coated for 40 seconds. The photos were captured at various magnifications.(JEOL FE-SEM IT800)

## X-Ray diffraction:

The X-ray diffraction (XRD) pattern was recorded by using a Bruker D8 Advance X-ray diffractometer using CuKα radiation (λ = 1.5406 Å), 40 kV- 40mA, 2θ/θ scanning mode. Data was collected for the 2θ range of 5 to 90 degrees with a 0.029649 degree step.

## Antimicrobial activity:

The agar well diffusion method was used to test the antibacterial properties of green synthesized copper oxide nanoparticles. Mueller Hinton agar plates have been prepared and sterilized in an autoclave at 121°C for 15-20 minutes. After sterilization, the medium was placed over the sterile Petri plate surface and left to cool to room temperature. Using sterile cotton swabs, the bacterial suspension (*Streptococcus mutans*, *Lactobacillus sp*, *Staphylococcus aureus* and *Candida albicans*) was equally distributed over the agar plates. A sterile polystyrene tip was used to make 9mm diameter wells in the agar plates. The wells were subsequently filled with various concentrations of CuO NPs (25 g, 50 g, and 100 g). As a control, an antibiotic (e.g Bacteria-Amoxyrite, Fungi- Fluconazole) was used. For fungal cultures, the plates were incubated at 37°C for 24 hours and 48 hours. The diameter of the inhibition zone surrounding the wells was measured to assess antibacterial activity. The diameter of the zone of inhibition was measured with a ruler, recorded in millimeters (mm), and the zone of inhibition was then calculated.

## Anti-oxidant activity of CuO nanoparticles:

## H2O2 assay:

The Ruch *et al*. technique was used to evaluate the extract's capacity to scavenge hydrogen peroxide (H2O2) [20]. 0.1 mL of extracts (25-400 g/mL) was put into eppendorf tubes, and their volume was increased to 0.4 mL with 50 mM phosphate buffer (pH 7.4) before 0.6 mL of H2O2 solution was added (2 mM). After being vortexed for 10 minutes, the reaction mixture's absorbance at 230 nm was assessed. The positive control in this experiment was ascorbic acid.

H2O2 activity (%) = *Abs (control)* - *Abs (sample)* *100 */Abs (control)*

Where, Abs (control): Absorbance of the control and Abs (test): Absorbance of the extracts/standard.

## Anti-inflammatory activity:

## Egg albumin denaturation assay:

To perform the Egg albumin denaturation experiment, 0.2mL of fresh egg albumin was combined with 2.8mL of phosphate buffer. The reaction mixture was supplemented with various amounts (10-50 g/mL) of P. Longum and P. Betle mediated Copper Oxide nanoparticles. The pH was set at 6.3. The sample was then maintained at room temperature for 10 minutes before being incubated in a water bath at 55°C for 30 minutes. The standard group consisted of diclofenac sodium, whereas the control group consisted of dimethyl sulphoxide. Following that, the samples were spectrophotometrically analyzed at 660nm.

Percentage of protein denaturation was determined utilizing following equation,

% inhibition = Absorbance of control - Absorbance of sample x 100 /Absorbance of control

## Results:

## Characterization of CuO nanoparticles:

## Visual observation:

**Figure 3:**
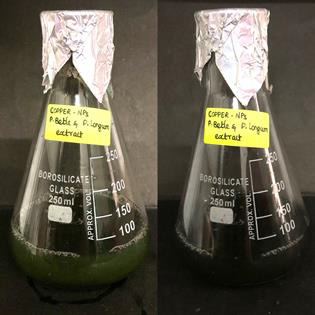
Shows a color shift from dark green to black, demonstrating the formation of copper nanoparticles.

Because of their unique optical characteristics, nanoparticles are quite interesting. They display a variety of colours during the synthesis process. The plant extract includes phytochemicals that convert copper sulphate into copper nanoparticles, as seen by the colour change. The transition of color from dark green to black indicates the synthesis of copper nanoparticles (Figure 3).

## UV-visible Spectroscopy:

**Figure 4:**
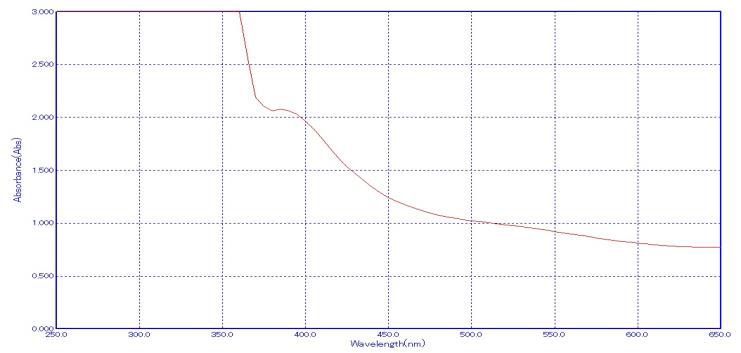
UV-Vis spectrophotometer analysis of synthesized CuO nanoparticles.

Copper nanoparticles were synthesized using copper sulphate and extracts of *Piper longum* and *Piper betle*, which had an absorbance peak at 350 nm (Figure 4). This peak was linked to the production of copper nanoparticles. The enlarged SPR peak found in the UV-visible spectrum indicated the formation of poly dispersed nano sized particles [21].

## SEM:

**Figure 5:**
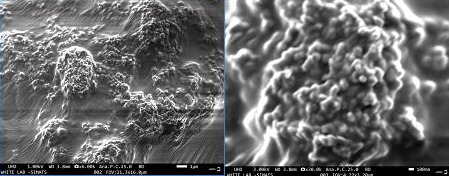
Scanning Electron Microscope, showing the shape of the CuO nanoparticles.

The CuO nanoparticles were examined at multiple magnifications like 30,000X and 6000X and the particles ranged in size from 30 to 90 nm. The image clearly shows the spherical shape of the CuO nanoparticles (Figure 5). The nanoparticles were clumped together because of the presence of bio-active compounds which are present in plant extract.

## FTIR:

**Figure 6:**
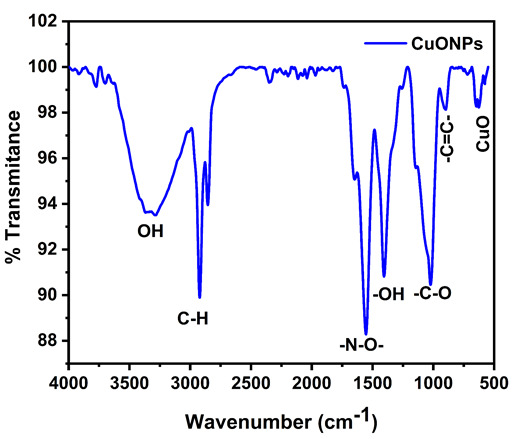
Shows the FT-IR Spectra of Copper Nanoparticles

FTIR measurements (Figure 6) were performed to detect potential biomolecules responsible for the reduction of Cu+ ions. The biomolecules that were selectively bound to the surface of the copper oxide nanoparticles were identified using FTIR spectroscopy. Figure 6 depicts the FTIR spectra of dried green synthesized CuO nanoparticles. The peaks are observed at 640 cm^-1^ indicating the CuO stretching vibrations, 893 cm^-1^ indicating the -C=C- Vinylidene stretching, 1029 cm^-1^ -C-O stretching in amino acid, 1402 cm^-1^ is identified as OH carboxylic acid, 1550 cm^-1^ as N-O Nitro stretching, 2349 cm^-1^ showed a slight peak level, 2853 cm^-1^ indicated as C-H in plane bending vibrations of alkenes, and 3374 cm^-1^ O-H stretch in the primary and secondary amide group.

## XRD:

**Figure 7:**
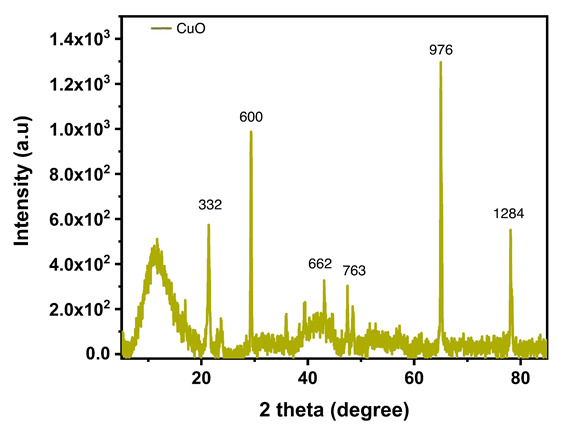
Shows the X-ray diffraction of synthesized CuNPs.

The crystalline structure of the synthesized nanoparticles was confirmed by the X-ray diffraction (XRD) peaks at 2θ values of 22.791°, 29.280°, 65.739°, and 78.167° corresponding to (332), (600), (976), and (1284) (Figure 7) planes which confirm the cubic lattice of copper (as per JCPDS, copper file no. 04-0836). The sharp and strong peaks observed indicate that the sample is having high crystalline quality. The sample has exceptional crystalline quality, which is indicated by the sharp, strong peaks that were found. There is no crystallinity impurities detected, indicating that the sample is of high purity. The smaller peaks at 43.089° and 48.210°, corresponding to (662) and (763), respectively, suggest the source material Cu2O.

## Antibacterial activity of copper nanoparticles against oral pathogens

**Figure 8:**
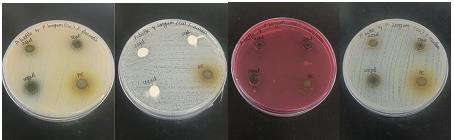
Antimicrobial activity of copper nanoparticles synthesized from *Piper betle* and *Piper longum*, formulation against *E. faecalis*, *S. Aureus*, *C. albicans*, and *S. mutans*.

**Figure 9:**
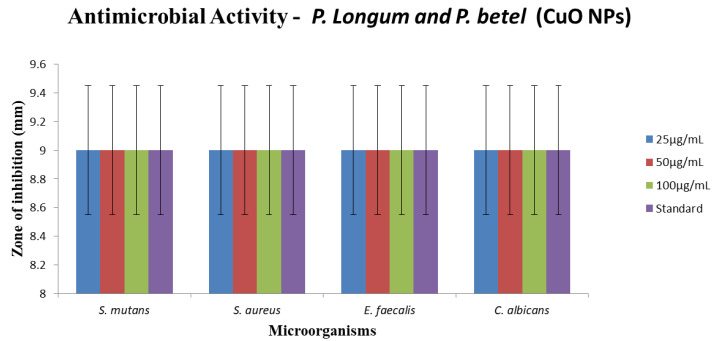
Graphical representation of antimicrobial activity of copper nanoparticles synthesized from Piper Longum and Piper Betle formulation against S.mutans, S,aureus,E.faecalis, C.albicans. S.aureus is having almost comparable antimicrobial activity as that of the control antibiotic disc.

Copper has remarkable antibacterial efficacy against a variety of oral infections, and its nanoparticle form enhances this property [22]. The zone of cell growth inhibition is generated by the disruption of the cell membrane by copper nanoparticles, which contributes to the disintegration of cell enzyme [23]. Our findings suggest that copper nanoparticles mediated by *Piper longum* and *Piper betle* have efficient antibacterial action comparable to the gold standards, with inhibition of 9 ± 0.5mm (Figure 8, Figure 9).

## Antioxidant Activity of CuO Nanoparticles:

**Figure 10:**
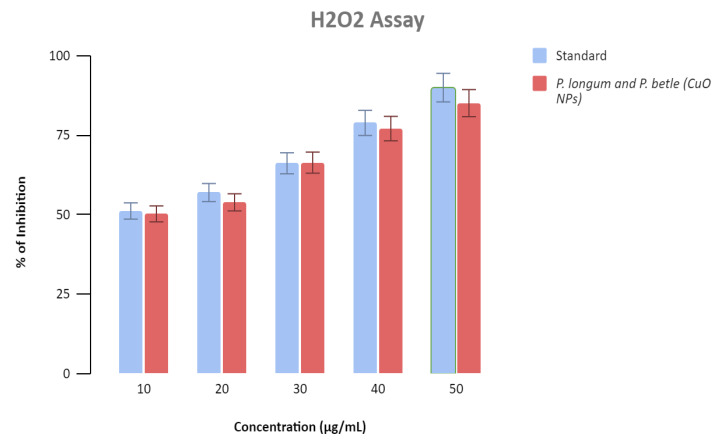
Graphical representation of AntiOxidant activity of copper nanoparticle synthesized from Piper Longum and Piper Betle formulation having comparable antioxidant activity to standard, Ascorbic Acid.

As indicated in Figure 10, a maximum of 85.16% activity inhibition was detected at 50 µg/ml and a minimum of 50.62% inhibition at the lowest dosage, 10 µg/ml. The IC50 for the produced CuNPs was 9.74 µg/ml, indicating that they are capable of scavenging 50% of the H2O2.

## Anti-inflammatory property of copper nanoparticles:

**Figure 11:**
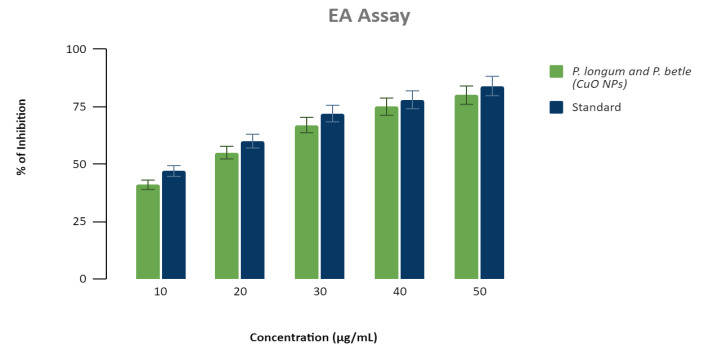
Anti Inflammatory Graphical representation of Anti-Inflammatory activity of copper nanoparticles synthesized from Piper Longum and Piper Betle formulation having comparable anti-inflammatory activity to standard Diclofenac Sodium.

The in vitro bioassay results of Green Synthesised-CuO NPs' anti-inflammatory properties against heat-induced egg albumin denaturation are reported in Figure 11. In a concentration-dependent manner, all tested doses greatly reduced denaturation of egg albumin. The maximal inhibition percentage was 81.13% at the highest tested concentration (50 µg/mL), while Diclofenac, the reference medication, inhibited at the same concentration by 84.4%.

## Discussion:

In today's scenario, Nano-technology has immense applications. Advances in nanotechnology are deeply intertwined with other technologies, many of which have received far greater attention. Copper has long been known for its antibacterial and anti-inflammatory effects [24]. The cost-effectiveness of the copper nanoparticles makes them a viable substitute for gold and silver nanoparticles. They can also be produced inexpensively [25]. Environmentally friendly copper nanoparticle production is now gaining popularity [26]. Due to the plant's ability to function as a capping and reducing agent as well as its eco-friendliness, green production of nanoparticles is becoming increasingly popular [26]. Species of the genus *Piper* are important medicinal plants used in various systems of medicine. They are used in traditional medicine, including the Ayurvedic system of medicine. Piper betle and Piper longum contain many phytoconstituents like alkaloids, tannins, glycosides, reducing sugars, and saponins [27]. *P. longum* has demonstrated remarkable effects against numerous diseases and conditions, including cancer, inflammation, depression, diabetes, obesity, and hepatotoxicity [28]. P.longum and P.betle are considered as reducing agents for synthesizing copper nanoparticles of different morphology because they are rich in polyphenols and other organic groups [29].

The reduction mechanism is carried out in two stages. Initially, when the precursor is introduced, a complex is created by breaking the -OH bond and creating a partial bond with a metal ion. The metal ions are subsequently reduced to nanoparticles due to the partial bond breaking, and these are then oxidized to ortho-quinone [30]. The color shift in this study shows the formation of copper nanoparticles, which is consistent with previous studies [31]. Then, UV-Vis spectroscopy was used to characterize the synthesized CuNPs, and a strong peak revealed the presence of CuNPs. The SEM picture revealed relative Spherical shape NPs with sizes ranging from 30 to 90nm. FT-IR spectroscopy analysis was done to determine the main components that contributed to the Cu+ reduction into CuNPs in the aqueous leaf extracts of P. Betle and P. Longum longum. FT-IR analysis revealed variations in the absorbance peak of CuNPs with various points ranging from 640 cm^-1^ to 3374 cm^-1^. This anticipates the possible functional groups that are involved in the reduction of Cu+ to CuNPs. The presence of copper nanoparticles is confirmed by the XRD peaks of synthesized CuNPs, which have 2θ values of 22.791°, 29.280°, 65.739°, and 78.167° corresponding to (332), (600), (976), and (1284) [32]. The peaks were then validated using the JCPDS database. The peaks observed from the XRD study clearly correlate to the cubic lattice of copper [33,34]. In this study, we used the agar well diffusion technique to test CuNPs for antibacterial activity against E. faecalis, S. aureus, S. mutans, and C. albicans. The present study's findings revealed average antimicrobial activity. According to the H2O2 assay, the synthesized CuNP's had a stronger antioxidant capability at low concentrations and was close to the gold standards at high concentrations [29,35].

## Conclusion:

In conclusion, this work proved the environmentally friendly, commercially feasible green synthesis of copper nanoparticles in the presence of copper sulphate using P. longum and P. betle leaf extracts as reducing agents. The early confirmation of the synthesis of CuNPs was the change in color of the solution, which was validated by UV-Vis spectroscopy. FTIR identified the presence of CuO nanostructures and functional groups in charge of reducing Cu+ ions. An X-ray diffractogram (XRD) analysis demonstrated that the produced nanoparticles at 2θ values of 22.791°, 29.280°, 65.739°, and 78.167° matched the planar cubic copper lattice. The scanning electron microscope was used to examine the morphology of the CuO nanoparticles. A completed antibacterial assay revealed that it effectively destroys the germs in a manner similar to the control against S. aureus S. mutant, E. coli, and C. albicans. The produced copper nanoparticles demonstrated strong antioxidant activity, according to the H2O2 test. According to the egg albumin assay CuO nanoparticles exhibited good anti-inflammatory properties. With all these qualities, biosynthesized CuNPs can be used in the future for the clinical development of medicinal drugs and protection against infectious illnesses.
